# Mitochondrial fission is an acute and adaptive response in injured motor neurons

**DOI:** 10.1038/srep28331

**Published:** 2016-06-20

**Authors:** Sumiko Kiryu-Seo, Hiromi Tamada, Yukina Kato, Katsura Yasuda, Naotada Ishihara, Masatoshi Nomura, Katsuyoshi Mihara, Hiroshi Kiyama

**Affiliations:** 1 Department of Functional Anatomy and Neuroscience, Graduate School of Medicine, Nagoya University, 65 Tsurumai-cho, Showa-ku, Nagoya 466-8550, Japan; 2 CREST, JST, 65 Tsurumai-cho, Showa-ku, Nagoya 466-8550, Japan; 3 Department of Protein Biochemistry, Institute of Life Science, Kurume University, Kurume 839-0864, Japan; 4 Department of Medicine and Bioregulatory Science, Graduate School of Medical Science, Kyushu University,Fukuoka 812-8382, Japan; 5 Department of Molecular Biology, Graduate School of Medical Science, Kyushu University, Fukuoka 812-8382, Japan

## Abstract

Successful recovery from neuronal damage requires a huge energy supply, which is provided by mitochondria. However, the physiological relevance of mitochondrial dynamics in damaged neurons *in vivo* is poorly understood. To address this issue, we established unique bacterial artificial chromosome transgenic (BAC Tg) mice, which develop and function normally, but in which neuronal injury induces labelling of mitochondria with green fluorescent protein (GFP) and expression of *cre* recombinase. GFP-labelled mitochondria in BAC Tg mice appear shorter in regenerating motor axons soon after nerve injury compared with mitochondria in non-injured axons, suggesting the importance of increased mitochondrial fission during the early phase of nerve regeneration. Crossing the BAC Tg mice with mice carrying a floxed dynamin-related protein 1 gene (*Drp1*), which is necessary for mitochondrial fission, ablates mitochondrial fission specifically in injured neurons. Injury-induced *Drp1*-deficient motor neurons show elongated or abnormally gigantic mitochondria, which have impaired membrane potential and axonal transport velocity during the early phase after injury, and eventually promote neuronal death. Our *in vivo* data suggest that acute and prominent mitochondrial fission during the early stage after nerve injury is an adaptive response and is involved in the maintenance of mitochondrial and neuronal integrity to prevent neurodegeneration.

For successful recovery from damage, the huge energy is required for the reprogramming of gene expression, reorganisation of intracellular signalling and glial cell interaction, such as with microglial cells and astrocytes^1^,^2^,^3^,^4^,^5^,^6^. Neurons control their mitochondrial dynamics to maintain Ca^2+^ buffering and to satisfy the energy demand. Our previous studies have demonstrated that mitochondrial integrity and dysfunction play pivotal roles in determining the fate of injured motor neurons^7^,^8^. In the last decade, multiple findings have suggested that the morphology and motility of neuronal mitochondria are associated with neuronal and axonal integrity^9^,^10^. In neurodegenerative diseases, mitochondria often show abnormal shape and irregular axonal transport in damaged neurons and axons prior to clinical onset^11^,^12^,^13^,^14^,^15^. However, it is unclear how mitochondrial dynamics contributes to axonal and neuronal integrity in damaged neurons *in vivo*.

Of the morphological changes that occur in injured neurons, the physiological relevance of mitochondrial fission remains to be determined^16^. Models for neurological diseases demonstrate acceleration of mitochondrial fission, subsequent synaptic dysfunction, neuronal damage and ultimately cell death, suggesting that mitochondrial fission is associated with cell death^17^,^18^,^19^. By contrast, *Drp1* (dynamin-related protein 1)-deficient mice, in which mitochondrial fission is suppressed in neurons, show severe abnormalities in developing and post-mitotic neurons, suggesting that fission could, in fact, be a positive regulator of neuronal survival^20^,^21^,^22^,^23^,^24^. Mitochondrial fission is also considered to be associated with axonal transport of mitochondria in highly polarised neurons. Neurons require transport of mitochondria to sites far from the soma, such as growth cones, axonal branching points and synaptic terminals, where local energy production is critical^25^,^26^,^27^,^28^,^29^,^30^. Defects in mitochondrial transport to these peripheral locations can cause local energy depletion and disruption of Ca^2+^ buffering, which can trigger synaptic dysfunction and loss. Although mitochondrial fission is crucial in neuronal development as well as maintenance, the significance of mitochondrial fission in neurons damaged by traumatic injury or neurodegenerative diseases is unclear.

Most studies of mitochondrial dynamics have been carried out *in vitro*, in which glial cells are missing and, therefore, represent a very different environment to that *in vivo*. The difference between *in vivo* and *in vitro* environments, therefore, confounds understanding of the functional significance of mitochondrial dynamics under neuronal stress. To further address mitochondrial dynamics *in vivo*, we have created a unique transgenic (Tg) mouse using bacterial artificial chromosome (BAC) technology in which, in response to nerve injury, mitochondria are labelled with GFP and *cre* recombinase is expressed. To ensure responsiveness to nerve injury, we employed a regulatory element of the activating transcription factor 3 (*Atf3*) gene, which we have characterised as a unique nerve injury-responsive transcription factor^3^,^31^,^32^,^33^. Using this BAC Tg mouse, we can investigate how mitochondria behave in response to nerve injury and how this behaviour influences neuronal status. Furthermore, *Drp1* ablation specifically in injured neurons demonstrates that mitochondrial fission at the early stage after injury is an important event for maintaining neuronal and mitochondrial integrity.

## Results

### BAC Tg mice with labelled mitochondria and cre expression in response to nerve injury

**Figure 1:**
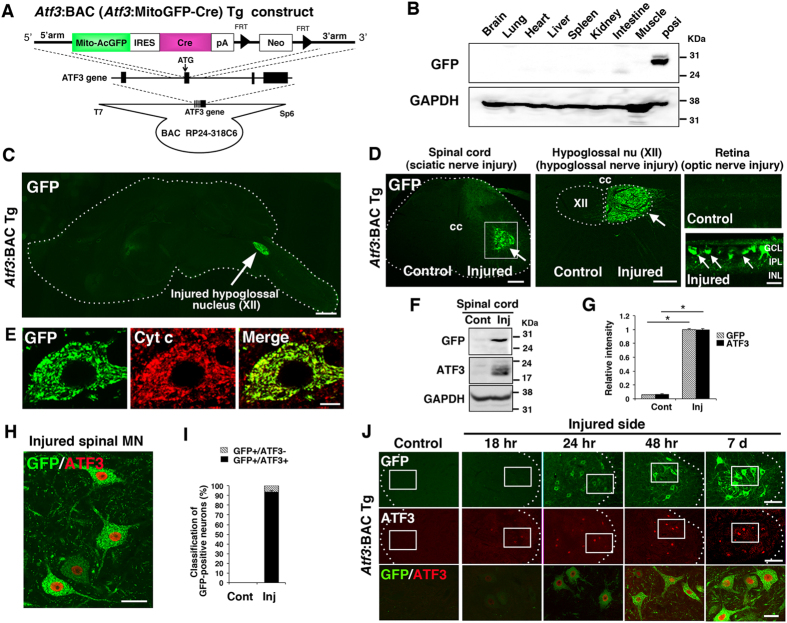
Expression of GFP in injury-inducible *Atf3*:BAC Tg mice. (**A**) Schematic of the shuttle vector used to generate *Atf3*:BAC Tg mice. The black boxes denote *Atf3* exons. Arrowheads indicate FRT sequence. (**B**) Immunoblot of GFP in various tissues obtained from the BAC Tg mice. The cell lysate of COS-7 cells over-expressing MitoGFP was used as a positive control. (**C**) The expression of GFP in sagittal brain sections from adult *Atf3*:BAC Tg mice. Dashed lines show the outline of the section. Arrow: injured hypoglossal nucleus at 7 days after hypoglossal nerve injury. (**D**) Immunohistochemical staining of GFP in spinal cord at 7 days after sciatic nerve injury (left panel), in medulla oblongata at 7 days after hypoglossal nerve injury (middle panel) and in retinae at 3 days after optic nerve injury (right panels). Arrows indicate injury-inducible expression of GFP signals. cc: central canal, XII; hypoglossal nucleus, GCL; retinal ganglion cell layer, IPL; inner plexiform layer, INL; inner nuclear layer. (**E**) The GFP signal was colocalized with cytochrome c (Cyt c) in injured spinal motor neuron. (**F**) Immunoblot of spinal cord lysate at control and at 7 days after sciatic nerve injury. (**G**) Band intensity of (**F**) was quantified and normalized against GAPDH. Values are mean ± SEM. (*p < 0.001, n = 3 independent experiments). (**H**) GFP-positive spinal motor neurons express ATF3 at 7 days after injury. (**I**) Percentage of ATF3-positive (GFP+/ATF3+) and ATF3-negative cells (GFP+/ATF3−) of all GFP-positive motor neurons in control and injured sides. Values are mean ± SEM (n = 4 mice). (**J**) The expression of GFP and ATF3 in injured motor neurons at 18 hours (18 hr), 24 hours (24 hr), 48 hours (48 hr) and 7 days (7 d) after sciatic nerve injury. Dashed outlines correspond to the boundary between gray and white matter. The area surrounded by a box is merged and magnified in lowest panel. Scale bars, 1000 μm in (**C**), 300 μm in (**D**, left), 200 μm in (**D**, middle), 40 μm in (**D**, right), 10 μm in (**E**), 100 μm in (**J**, upper) and 30 μm in (**H,J**, lowest).

To visualise injury-inducible mitochondrial dynamics, we produced Tg mice using a BAC clone (RP24-318C6) bearing ~75 kb of 5′ and ~85 kb of 3′ sequence flanking the activating transcription factor-3 gene (*Atf3*) because *Atf3* expression is specifically and substantially induced in response to nerve injury (Supplementary Fig. S1)^32^. The start codon of *Atf3* and the 3′ end of the same exon were replaced by a fragment carrying mitochondria-targeted green fluorescent protein from *Aequorea coerulescens* (MitoAcGFP), followed by an internal ribosomal entry site, *cre* recombinase and a poly-adenylation signal sequence (Fig. 1A). Western blotting showed no obvious GFP signal in various tissue samples of *Atf3*:BAC Tg (*Atf3*:MitoAcGFP-Cre) mice (Fig. 1B). An immunohistochemical study of sagittal brain sections showed a negligible level of GFP expression throughout the brain. However, in hypoglossal nerve-injured mice, intense GFP expression was observed in the hypoglossal nucleus (Fig. 1C). In peripheral nervous system injury models, sciatic nerve injury induced the expression of GFP in spinal motor neurons in the ventral horn of the nerve-injured side, and unilateral hypoglossal nerve injury induced GFP expression in hypoglossal motor neurons of the injured side (Fig. 1D, Supplementary Fig. S2). Similarly, optic nerve injury, which is a CNS injury model, resulted in prominent GFP expression in injured retinal ganglion cells (Fig. 1D, Supplementary Fig. S2). The localisation of GFP completely overlapped with cytochrome c, a mitochondrial marker, because of the mitochondrial targeting signal attached to GFP (Fig. 1E).

**Figure 2:**
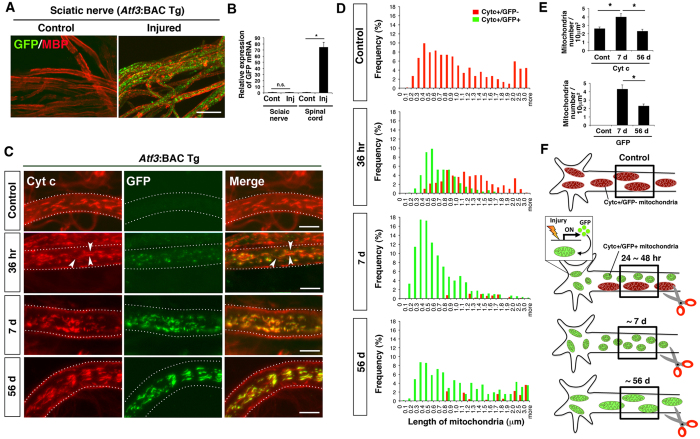
The length of axonal mitochondria was changed after sciatic nerve injury. (**A**) Immunohistochemical staining for GFP and myelin basic protein (MBP) using teased sciatic nerve of *Atf3*:BAC Tg mice at 7 days after injury. (**B**) The expression of GFP mRNA in control and injured sides of sciatic nerves and spinal cord. Data are the mean ± SEM, compared with that of control side. (n = 3 independent experiments, n.s. not significant, *p < 0.001). (**C**) High magnification of a single sciatic nerve axon stained with cytochrome c (Cyt c) and GFP. The mitochondrial morphology changes over control, 36 hr, 7 d and 56 d after sciatic nerve injury. Arrowheads indicate GFP-negative and cytochrome c-positive mitochondria, which are considered to be pre-existing in the axon before injury. Dashed lines show the outline of a single axon. (**D**) Frequency distribution of axonal mitochondrial length after sciatic nerve injury. The red bars show GFP-negative and Cyt c-positive mitochondria, while the green bars show GFP-positive and Cyt c-positive mitochondria. Data was obtained from control (996 mitochondria in six mice), 36 hr (746 mitochondria in six mice), 7 d (1353 mitochondria in six mice) and 56 d (451 mitochondria in four mice) after sciatic nerve injury. (**E**) The number of Cyt c-stained and GFP-labelled mitochondria per 10 μm^2^ area in sciatic nerve was counted. Data represent means ± SEM. *p < 0.001, n = 4–6 mice. (**F**) A schematic illustration of axonal mitochondria before and after nerve injury. Red mitochondria indicate pre-existing mitochondria before nerve injury, while green mitochondria indicate newly labeled mitochondria in soma after nerve injury. The boxed area of the proximal axon is shown in Fig. 2C. 36 hr, 7 d and 56 d; 36 hours, 7 and 56 days after nerve injury respectively. Scale bars, 20 μm in (**A**) and 5 μm in (**C**).

Western blot analysis showed that the protein levels of both exogenous GFP and endogenous ATF3, an injury-inducible transcription factor, were induced in the spinal cord in response to sciatic nerve injury (Fig. 1F,G). In line with this, immunohistochemistry showed that most GFP-positive cells in the spinal cord expressed ATF3 in the nucleus (Fig. 1H,I). A few GFP-positive neurons did not express ATF3, probably because the nuclei of these ATF3-negative cells were not present in the tissue section. We also examined whether the expression of GFP in spinal motor neurons was induced in a similar time course to that of ATF3 following sciatic nerve injury (Fig. 1J). GFP- and ATF3-immunoreactive signals were very faint in spinal motor neurons 18 h after injury. Twenty-four hours after nerve injury, ATF3 and GFP immunoreactivity became evident and were increased at 48 h after injury. Thereafter, GFP expression became prominent in dendrites and axons in addition to soma at 7 days after injury, when hundreds of genes are up- and down-regulated in injured motor neurons^6^,^32^. The expression of GFP was still observed at 56 days after injury, when nerve regeneration was completed (see also Fig. 2C). These data indicate that the injury-induced expression of mitochondria-targeted GFP is successfully regulated by the *Atf3* regulatory element in the BAC transgene.

### Mitochondrial length decreases in axons following nerve injury

Using the *Atf3*:BAC Tg mouse, we examined GFP-labelled mitochondria in axons before and after sciatic nerve injury. For the observation of whole individual mitochondria in axons, the whole mount immunohistochemical approach using a teased fibre, rather than ordinary immunohistochemical staining using tissue sections, is advantageous because tissue sectioning results in sectioned mitochondria (Supplementary Fig. S3). Using the whole mount technique, we observed marked dot-like GFP signals in proximal regions of injured sciatic nerves, while no GFP signal was present in control nerves (Fig. 2A). Quantitative RT-PCR showed that GFP mRNA levels were markedly increased in nerve-injured spinal cord, while Schwann cells in injured nerves did not express GFP mRNA (Fig. 2B). Proteins with a mitochondrial targeting signal are rapidly imported into mitochondria after translation in the soma and are not observed in other organelles^34^,^35^. Therefore, mitochondria in the soma of *Atf3*:BAC Tg mice are labelled by GFP in response to nerve injury and are transported into the axon. These results, together with those in Fig. 1, indicate that *Atf3*:BAC Tg mice can be used to monitor mitochondria in injured neurons and axons.

We examined the length of individual mitochondria in axons before and after sciatic nerve injury (Fig. 2C). In the control nerve, all mitochondria were cytochrome c positive but GFP negative and were relatively longer. At the early stage, 24–48 h after nerve injury, GFP-labelled mitochondria were already apparent in the soma of injured motor neurons (Fig. 1J), and cytochrome c-/GFP double-positive mitochondria appeared shorter in injured axons. At this time point, GFP-negative mitochondria were sill observed in injured axons (Fig. 2C,D,F). Seven days after nerve injury, when changes to intracellular metabolism reach a peak level^6^,^32^, almost all mitochondria were labelled by GFP and were much shorter than those in the control nerve. Thereafter, the short GFP-labelled mitochondria gradually lengthened and returned to the control length 56 days after nerve injury, when nerve regeneration was almost complete. Indeed, the length of mitochondria returned to the control length long before the completion of nerve regeneration (Supplementary Fig. S3). In addition, the number of fragmented mitochondria per unit area of an axon was also significantly increased at 7 days after nerve injury, and returned to the basal level at 56 days after injury (Fig. 2E). This finding suggests that mitochondrial fission is increased in damaged motor neurons during the first 7 days after injury, although the protein levels of fission and fusion-related proteins, DRP1, Mitofusin-1 (MFN1) and MFN2, in motor neurons did not differ between before and after nerve injury (Supplementary Fig. S4).

### Successful Drp1 deletion in response to nerve injury

**Figure 3:**
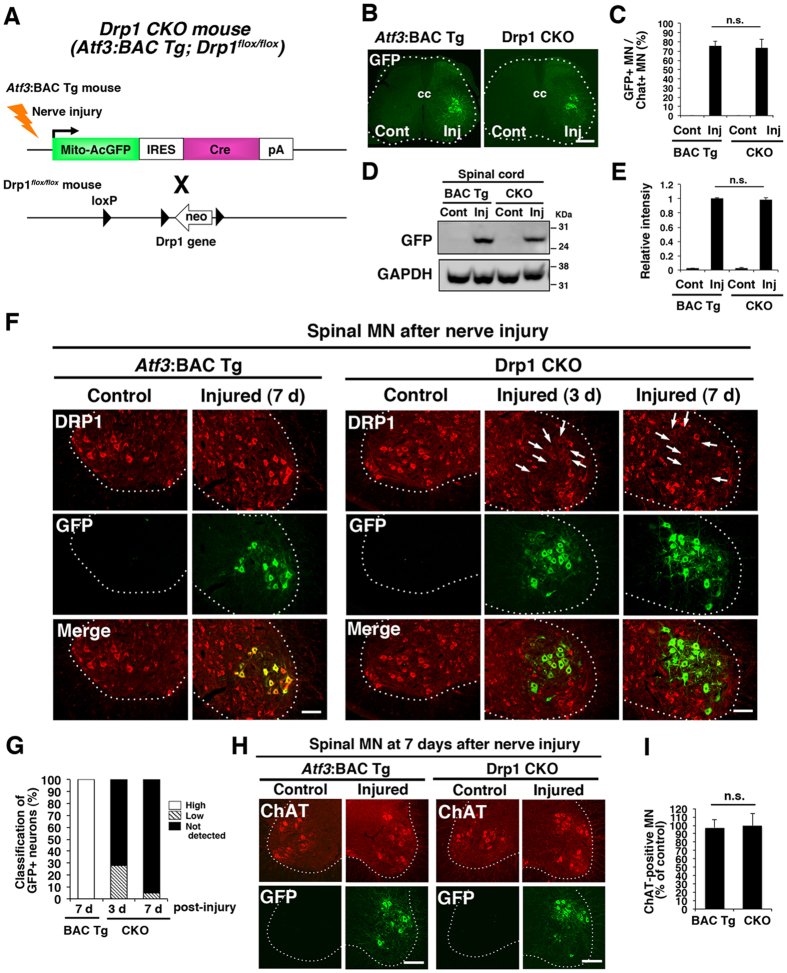
Ablation of *Drp1* specifically in injured motor neurons. (**A**) Schematic illustration of the generation of injury-inducible *Drp1* conditional knockout (Drp1 CKO) mice. (**B**) The immunohistochemical staining of GFP in spinal motor neurons at 7 days after sciatic nerve injury in *Atf3*:BAC Tg and Drp1 CKO mice. (**C**) The number of GFP-positive neurons among choline acetyltransferase (ChAT) positive motor neurons was counted. Data are the mean ± SEM (n = 4 mice, n.s. not significant). (**D**) Immunoblot using the spinal cord tissues from control and injured sides of both *Atf3*:BAC Tg and Drp1 CKO mice at 7 days after sciatic nerve injury. (**E**) Band intensity of (**D**) was quantified and normalized against GAPDH. Values are the mean ± SEM. (*p < 0.001, n = 3 independent experiments). (**F**) Endogenous expression of DRP1 after sciatic nerve injury. Note that GFP-positive injured motor neurons lose DRP1 positive signals in Drp1 CKO mouse (arrows). (**G**) Classification of DRP1-expressing cells. GFP-positive injured motor neurons were categorized as “high” (more than 80% intensity of DRP1 protein compared with that in control motor neurons), “low” (20–50% intensity compared with that in control motor neurons) and “not detectable”(less than 20% intensity compared with that in control motor neurons). (n = 5 mice for each group) (**H**) The immunohistochemical staining of ChAT and GFP in spinal motor neurons at 7 days after sciatic nerve injury. Dashed outlines showed the boundary between gray and white matters. (**I**) Percentage of ChAT -positive surviving motor neurons on the injured side compared with the control side at 7 days after sciatic nerve injury. Data are the mean ± SEM. n = 5 mice per group, *p < 0.001.BAC Tg; *Atf3*: BAC Tg mice, CKO; Drp1 conditional knockout mice, Scale bars, 300 μm in (**B**), 100 μm in (**F**) and (**H**).

To evaluate the functional relevance of mitochondrial fission in damaged motor neurons during the first 7 days after injury, we crossed *Atf3*:BAC Tg (*Atf3*:MitoAcGFP-Cre) mice with floxed *Drp1* (Drp1^*flox/flox*^) mice to produce *Drp1* conditional knockout (Drp1 CKO) mice (Fig. 3A). In Drp1 CKO mice, *Drp1* is expected to be ablated in an injury-responsive manner because cre recombinase is induced under the control of *Atf3* regulatory elements. Drp1 CKO mice showed similar numbers of GFP-positive motor neurons (Fig. 3B,C) and similar amounts of exogenous GFP protein compared with control *Atf3*:BAC Tg mice in response to sciatic nerve injury (Fig. 3D,E). The quantification of DRP1 protein using western blotting was not carried out because non-injured neurons and glial cells express DRP1 in Drp1 CKO mice. Instead, we measured signal intensity of DRP1 immunoreactivity in control and injured motor neurons using spinal cord sections. We confirmed that DRP1 protein in GFP-positive injured motor neurons was reduced at 3 days after injury and was absent in GFP-expressing motor neurons of Drp1 CKO mice at 7 days after injury (Fig. 3F,G). The decrease of DRP1 protein levels was not caused by the death of injured motor neurons because almost all motor neurons, identified by the motor neuron marker choline acetyltransferase (ChAT), in both *Atf3*:BAC Tg and Drp1 CKO mice, survived the first 7 days after nerve injury (97.2 ± 3.6% in *Atf3*:BAC Tg n = 99.8 ± 5.8% Drp1 CKO, n = 3 mice for each group) (Fig. 3H,I). The level of MFN2 protein, which was abundant in motor neurons, was not different between the injured motor neurons of *Atf3*:BAC Tg and those of Drp1 CKO mice (Supplementary Fig. S5). Furthermore, *Drp1* was not ablated in Schwann cells or macrophages of nerve-injured Drp1 CKO mice, indicating that the mitochondrial phenotype was caused in an injured neuron autonomous manner (Supplementary Fig. S5C,D).

### Injury-induced Drp1-deficient axons show altered mitochondrial morphology

**Figure 4:**
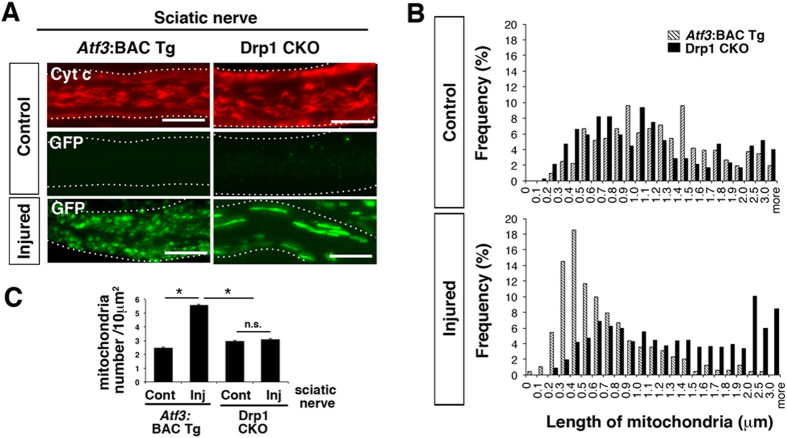
Morphological appearance of mitochondria in axons of *Atf3*:BAC Tg and Drp1 CKO mice. (**A**) Representative immunohistochemical staining of Cyt c and GFP in single axon of sciatic nerves in *Atf3*:BAC Tg and Drp1 CKO mice at 7 days after injury. (**B**) Frequency distribution of Cyt c- and GFP- stained mitochondrial length in control and injured nerves, respectively, using *Atf3*:BAC Tg (375 mitochondria in four mice for control side, 483 mitochondria in four mice for injured side) and Drp1 CKO mice (406 mitochondria in four mice for control side, 1057 mitochondria in seven mice for injured side) at 7 days after sciatic nerve injury. (**C**) Number of mitochondria per 10 μm^2^ of axons was counted at 7 days after injury in *Atf3*:BAC Tg (n = 4–6 mice) and Drp1 CKO mice (n = 4–7 mice). Data are means ± SEM. *p < 0.001. BAC Tg; *Atf3*:BAC Tg mice, CKO; Drp1 conditional knockout mice, Scale bars, 5 μm in (**A**).

First, we compared the morphological appearance of axonal mitochondria between *Atf3*:BAC Tg and Drp1 CKO mice (Fig. 4A). No difference was observed in the length of axonal mitochondria between BAC Tg and Drp1 CKO mice under normal conditions (Fig. 4B). However, a significantly larger number of GFP-labelled longer mitochondria (more than 2.5 μm in length) was observed in *Drp1*-deficient injured axons at 7 days after sciatic nerve injury (Fig. 4B). Furthermore, the number of axonal mitochondria per area was not increased in the injured axons of Drp1 CKO mice compared with that in *Atf3*:BAC Tg mice (Figs 2E and 4C).

### Drp1 deficiency affects mitochondrial quality at 7 days after nerve injury

**Figure 5:**
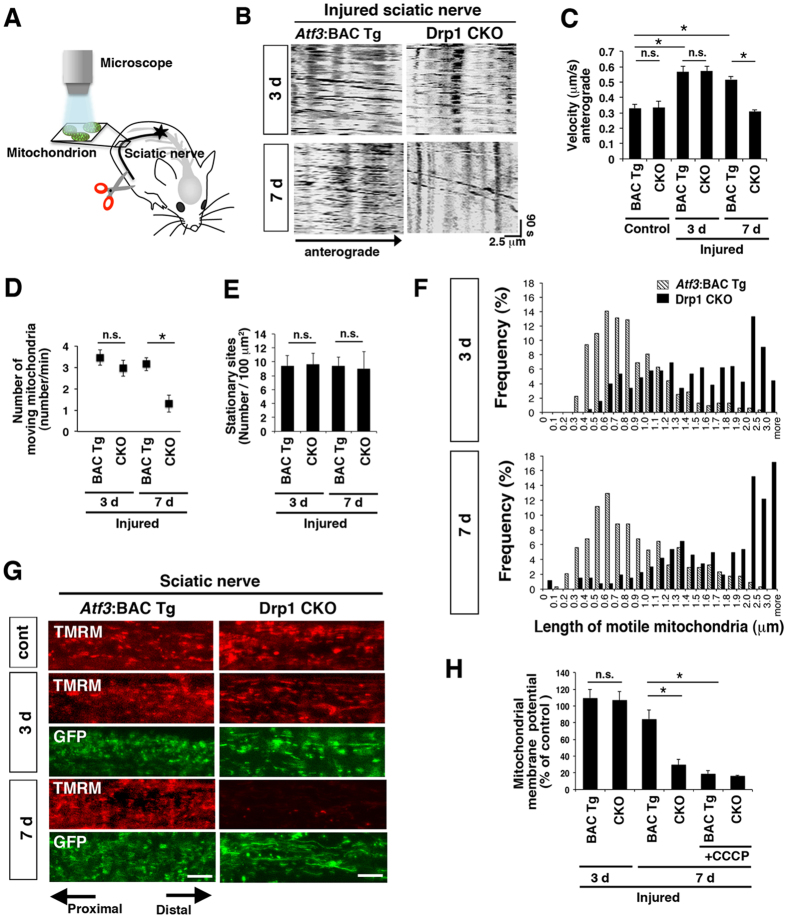
Alteration of mitochondrial quality in *Drp1-*deficient injured axons during 7 days after nerve injury. (**A**) Schematic drawing for *in vivo* time-lapse imaging of axonal mitochondria using sciatic nerve. (**B**) Representative kymograph of GFP-labelled mitochondrial transport in injured sciatic nerve *in vivo*. (**C**) Transport velocity of axonal mitochondria. Data are means ± SEM. *p < 0.001, analyzed by one-way ANOVA followed by Turkey’s post hoc test, *Atf3*:BAC Tg mice (BAC Tg): 0.33 ± 0.08 μm/s, 0.57 ± 0.09 and 0.52 ± 0.09 μm/s at control, 3 days (3d) and 7 days (7d) respectively, Drp1 CKO: 0.34 ± 0.12 μm/s, 0.57 ± 0.09 and 0.31 ± 0.08 μm/s at control, 3 and 7 days respectively, n = 4–6 mice in each group. Mitochondria in control nerves were labeled by TMRM before measurement. (**D**) The number of GFP-labelled mitochondria transported in a single axon at 3 and 7 days after sciatic nerve injury. Data are means ± SEM. (n = 4 mice, *p < 0.001). (**E**) The number of stationary mitochondrial sites per 100 μm^2^ area in sciatic nerve. Values are means ± SEM. (n = 4 mice, n.s. not significant). (**F**) Frequency distribution of the length of mobile GFP-labeled mitochondria in control and injured sciatic nerves of *Atf3*:BAC Tg mice (275 mitochondria in four mice at 3 days, 349 mitochondria in four mice at 7 days) and Drp1 CKO mice (250 mitochondria in four mice at 3 days, 409 mitochondria in five mice at 7 days) after injury. (**G**) Representative images of membrane potential sensing dye, TMRM (red)- and GFP (green)-labelled mitochondria in injured axons. (**H**) Percentage of mitochondrial membrane potential in injured nerves, compared with that in control sciatic nerves at 3 days (n = 3–5 mice in each group, mean ± SEM. *p < 0.001, ANOVA Turkey’s post hoc test). BAC Tg; *Atf3*:BAC Tg mice, CKO; Drp1 conditional knockout mice. Scale bars, 5 μm in (**G**).

Next, we clarified how changes to mitochondrial morphology are linked to mitochondrial integrity during the first 7 days after injury when injured neurons of both *Atf3*:BAC Tg and Drp1 CKO mice still looked healthy. We performed *in vivo* time-lapse imaging to monitor axonal transport and membrane potential of mitochondria in control and injured sciatic nerves of both mice (Fig. 5A).

We used kymograph to identify motile and stationary mitochondria (Fig. 5B). As shown in Fig. 5C, mitochondrial transport velocity in normal sciatic nerves was approximately 0.3 μm/s in both *Atf3*:BAC Tg and Drp1 CKO mice, which was in agreement with previous studies^36^. Three days after injury, transport velocity of mitochondria in injured axons of *Atf3*:BAC Tg and Drp1 CKO mice was increased compared with that in control nerves of both mice. However, the transport velocity of GFP-labelled mitochondria was then significantly decreased in *Drp1*-deficient axons at 7 days after injury, compared with that in *Atf3*:BAC Tg axons (Fig. 5C and Supplementary movies S1–S4). Consistent with this, mitochondrial motility was similar in BAC Tg and Drp1 CKO mice during the first 3 days after injury, but was significantly down-regulated in Drp1 CKO mice at 7 days after injury (Fig. 5D and Supplementary Fig. S6A). The stationary mitochondrial sites per area were unchanged between *Atf3*:BAC Tg and Drp1 CKO mice before and after nerve injury (Fig. 5E and Supplementary Fig. S6B). The data were further supported by unchanged expression level of syntaphilin, which anchors mitochondria at stop sites in axons^30^, between *Atf3*:BAC Tg and Drp1 CKO mice, both before and after nerve injury (Supplementary Fig. S6C). It is well established that two types of mitochondrial pools exist in axons; one third of mitochondria are motile, while the remainder are stationary^37^. In general, motile mitochondria are shorter than stationary mitochondria^38^. We wondered whether a small number of shorter mitochondria were motile in *Drp1*-deficient injured axons or whether the majority of longer mitochondria were motile (Fig. 4B). To address this point, we measured the length of motile mitochondria using time-lapse images (Fig. 5F). As we expected, motile mitochondria were short in injured axons of BAC Tg mice. However, the increased number of motile mitochondria was longer in *Drp1*-deficient injured axons both at 3 and 7 days after injury (Fig. 5F). The transport velocity and motility of the longer mitochondria were declined in *Drp1*-deficient axons at 7 days after injury, as shown in Fig. 5B–D. These findings demonstrate that even *Drp1*-deficient longer mitochondria can be motile and transported in a similar way to shorter mitochondria in *Atf3*:BAC Tg mice during at least the first 3 days after injury; longer mitochondria then gradually lose this ability and it is lost by 7 days after injury.

To further examine the quality of mitochondria after nerve injury, we applied a membrane potential sensing dye, tetramethylrhodamine methylester (TMRM), to control and injured sciatic nerves of both mice and monitored signal intensity of TMRM in axonal mitochondria (Fig. 5G,H). The mitochondrial membrane potential was maintained in the injured nerve of both control BAC Tg and Drp1 CKO mice at 3 days after injury. Seven days after injury, the membrane potential was markedly suppressed in the Drp1 CKO mouse, but not in the control BAC Tg mouse. Treatment with a mitochondrial uncoupling reagent, carbonyl cyanide *m*-chlorophenyl hydrazine (CCCP), confirmed complete suppression of mitochondrial membrane potential in both BAC Tg and Drp1 CKO mice (Fig. 5G,H). Collectively, these findings indicate that Drp1 CKO mice are able to maintain mitochondrial quality, such as transport velocity and membrane potential at early stages after injury, at least for the first 3 days; however, mitochondrial quality then decreases along with the increasing energy requirements of nerve repair.

### Injury-induced Drp1 deletion alters the morphological appearance of mitochondria in soma

**Figure 6:**
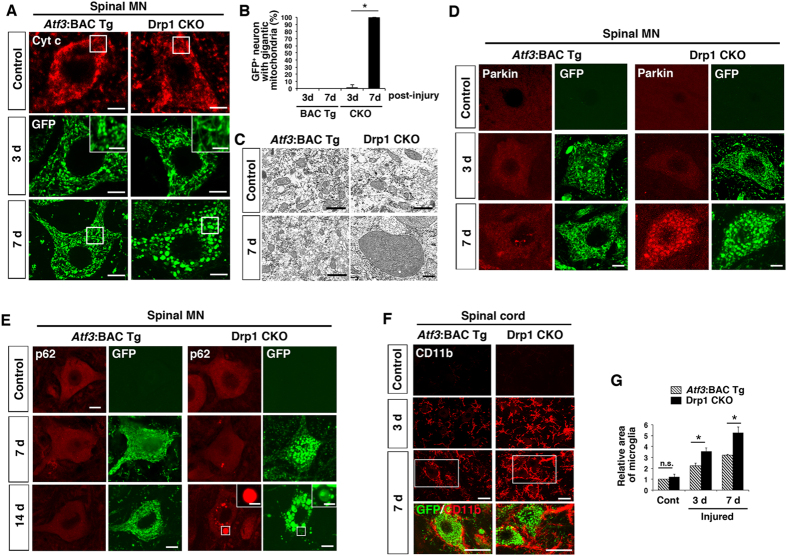
Injury-inducible *Drp1*-deficient motor neurons affect mitochondrial and neuronal integrity during first 7 days after injury. (**A**) The immunohistochemical staining of mitochondria using Cyt c antibody for control motor neurons and GFP antibody for injured motor neurons. High magnification of mitochondria at 3 days after injury is shown in insets. The area surrounded by a box is magnified in (**C**). (**B**) The percent ratio of GFP-positive cells with spherical and gigantic mitochondria after nerve injury. Values are mean ± SEM (n = 4 mice for each group, *p < 0.001). (**C**) Electron micrograph of mitochondria in motor neurons of *Atf3*:BAC Tg and Drp1 CKO mice using x 12,000 (excluding injured side of Drp1 CKO) and x 8,000 (injured side of Drp1 CKO) objectives. (**D**) Immunohistochemical staining of Parkin and GFP in spinal motor neurons of *Atf3*:BAC Tg and Drp1 CKO mice at control, 3 d and 7 d after sciatic nerve injury. (**E**) Immunostaining of p62/SQSTM1 and GFP in injured spinal motor neurons at control, 7 d and 14 d after sciatic nerve injury. Asterisk denotes mitochondrion magnified in inset. (**F**) Immunostaining of CD11b and GFP in injured lumbar spinal cord at 3 d and 7 d after sciatic nerve injury. The area surrounded by a white box is magnified in lowest panel. (**G**) Relative area occupied by microglia in injured spinal cord. Total area of microglial cells in 150 μm^2^ was quantified. Values are mean ± SEM (n = 4 mice for each group, *p < 0.001). 3 d, 7 d and 14 d; 3, 7 and 14 days after nerve injury respectively. BAC Tg; *Atf3*:BAC Tg mice, CKO; Drp1 conditional knockout mice, Scale bars, 5 μm in (**A**), 3 μm (**A** and **E**, inset), 1 μm in (**C**), 10 μm in (**D,E**) and 30 μm in (**F**).

We examined further the morphological appearance of mitochondria in the soma of motor neurons at 7 days after injury. The morphology of cytochrome c-stained mitochondria in motor neurons of the control side appeared the same between *Atf3*:BAC Tg and Drp1 CKO mice. However, GFP-labelled mitochondria in the soma of nerve-injured Drp1 CKO motor neurons became tubular and interconnected at 3 days after injury in contrast to those in control BAC Tg mice (Fig. 6A). Intriguingly, after this time point, the morphology of mitochondria in all the injured motor neurons of Drp1 CKO mice switched to being abnormally round and gigantic with compacted cristae (Fig. 6A–C, Supplementary Fig. S6D).

The immunoreactivity of Parkin, which is recruited to mitochondria in the event of membrane potential loss^39^, was not apparent at 3 days after injury in either mouse. Thereafter, prominent accumulation of Parkin was observed on almost all huge and round mitochondria in Drp1 CKO mice, whereas it was not apparent on mitochondria in *Atf3*:BAC Tg mice (Fig. 6D). This suggests that mitochondria in the soma of Drp1 CKO mice suffer a severe loss of membrane potential at approximately 7 days after nerve injury when a massive energy supply is required. The time course of decreased mitochondrial quality in the soma of *Drp1*-deficient injured motor neurons was consistent with that in *Drp1-*deficient injured axons shown in Fig. 5. The Parkin-mediated degradation system is tightly associated with autophagy; therefore, we examined the expression of p62/SQSTM1 (Fig. 6E). Injured motor neurons of both *Atf3*:BAC Tg and Drp1 CKO mice at 7 days after injury showed diffuse p62/SQSTM1 staining in cytoplasm. In the Drp1 CKO mouse, there were a few accumulations of p62 in the cytoplasm, which did not overlap with the huge spherical mitochondria. Thereafter, some gigantic mitochondria overlapped with p62 in dying motor neurons at 14 days after injury. These data suggest that the mitochondrial degradation system in *Drp1*-deficient injured motor neurons was deteriorated at 7–14 days after injury and that the autophagy system was partly involved in mitochondrial quality control in injured motor neurons.

A question that arises here is whether mitochondrial fission, which is initiated at an early stage after nerve injury (Fig. 2) actually affects injured motor neurons at 3 days after injury. To clarify the impact of *Drp1* deficiency during the first 3 days after injury, we compared the microglial phenotype of the injured side of the spinal cord between *Atf3*:BAC Tg and Drp1 CKO mice at 3 days after injury because our group and others have demonstrated that microglial behaviour plays a pivotal role at the early stage after injury in the fate of damaged motor neurons^6^,^32^,^40^,^41^. Immunohistochemistry using CD11b, which is associated with microglial activation, produced a much more intense signal in the proximity of injured motor neurons in Drp1 CKO mice compared with that in BAC Tg mice (Fig. 6F,G). Thus, *Drp1* deficiency in injured motor neurons affects neuronal status from an early stage to cause microglial hyper-activation, even when mitochondrial integrity seems to be in the normal physiological range.

### Drp1 deficiency in injured neurons accelerates neuronal death and axonal degeneration

**Figure 7:**
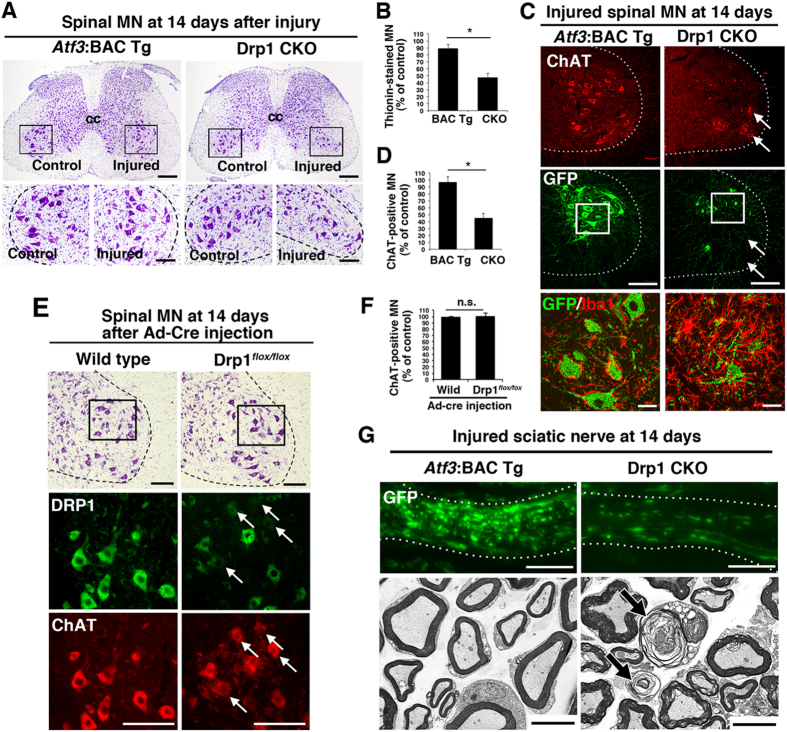
Injury-induced prevention of mitochondrial fission leads to neuronal death and axonal degeneration at 14 days after nerve injury. (**A**) Thionin staining of spinal cord sections in *Atf3*:BAC Tg and Drp1 CKO mice at 14 days after sciatic nerve injury. The area surrounded by a black box in the upper panel is magnified in the lower panel. (**B**) Percentage of thionin-stained surviving motor neurons at 14 days after sciatic nerve injury. Results represent the percent ratio of surviving motor neurons on the injured side compared with the contralateral side. Data are the mean ± SEM. n = 8 mice per group, *p < 0.001. (**C**) Immunostaining of injured spinal motor neurons for ChAT (red) and GFP (green). Note the presence of GFP-negative motor neurons (arrows). The area surrounded by a box is magnified in lowest panel showing that microglial activation (Iba1, red) in the proximity of injured motor neurons (GFP, green). (**D**) Percentage of ChAT-positive surviving motor neurons on the injured side compared with the control side at 14 days after sciatic nerve injury. Data are the mean ± SEM. n = 8 mice per group, *p < 0.001. (**E**) Thionin staining of spinal motor neurons at 14 days after injection of adenovirus carrying cre recombinase (Ad-Cre) in wild-type and Drp1^*flox/flox*^ mice (highest panel). The area surrounded by a box in highest panel was shown by immunostaining of DRP1 and ChAT in lower panels. Arrows indicate the reduced expression of DRP1 in ChAT-positive motor neurons. (**F**) Percentage of ChAT-positive motor neurons in wild-type and Drp1^*flox/flox*^ mice at 14 days after injection of Ad-Cre. Data are means ± SEM. n = 4 mice per group, *p < 0.001. (**G**) Immunohistochemical staining of GFP (upper) and electron micrograph using x 3,000 objective of injured sciatic nerve (lower) in *Atf3*:BAC Tg and Drp1 CKO mice at 14 days after nerve injury. Arrows indicate degenerating axons. BAC Tg; *Atf3*:BAC Tg mice, CKO; Drp1 conditional knockout mice, Scale bars, 250 μm in (**A**, upper), 100 μm in (**A**, lower; **C**, middle; **E**), 30 μm in (**C**, lowest) and 5 μm in (**G**).

Because the morphological appearance and quality of the mitochondria were markedly changed at 7 days after nerve injury, when injured neurons still looked healthy, *Drp1* deficiency is likely to affect the fate of nerve-injured motor neurons. We, therefore, examined the destiny of injured spinal motor neurons in Drp1 CKO mice at 14 days after sciatic nerve injury. At this time, the number of thionine-stained motor neurons was markedly decreased in Drp1 CKO mice, while *Atf3*:BAC Tg mice showed a survival rate of almost 100% (Fig. 7A,B). The cell death of injured motor neurons in Drp1 CKO1 mice was further confirmed using another peripheral nervous system injury model, the hypoglossal nerve injury model (Fig. S7A,B). To exclude over-estimation of surviving motor neurons by counting non-motor neurons, we also examined ChAT immunoreactivity (Fig. 7C,D), and obtained similar results to those using thionin staining. The survival of ChAT-positive motor neurons of Drp1 CKO mice decreased markedly, while *Atf3*:BAC Tg mice showed an almost 100% survival rate. The graph shown in Fig. 7D demonstrates that around 40% of ChAT-positive motor neurons in Drp1 CKO mice remained even at 14 days after nerve injury; however, these neurons were GFP negative, indicating non-injured neurons (Fig. 7C). In addition, in *Atf3*:BAC Tg mice, microglial cells became flat and adhered to the injured motor neurons, whereas microglial cells in Drp1 CKO mice were highly activated and not tightly adherent to the injured neurons, suggesting that they were phagocytosing the degenerating motor neurons (Fig. 7C). No significant difference was observed for other glial cells, such as astrocytes, between *Atf3*:BAC Tg and Drp1 CKO mice (Supplementary Fig. S7). To exclude the possibility that *Drp1* deficiency affects the survival of motor neurons in an injury-independent manner, we injected adenovirus carrying *cre* recombinase into the intact sciatic nerve of Drp1^*flox/flox*^ mice to ablate *Drp1* in motor neurons without nerve injury. At 14 days after virus injection, *Drp1*-deficient motor neurons without injury appeared healthy (Fig. 7E,F), suggesting that injury-induced acute mitochondrial fission is crucial for the adaptive response of injured motor neurons. Together with the degeneration of motor neuron cell bodies, electron microscopy observation of injured axons in Drp1 CKO mice showed characteristic degeneration morphology at 14 days after nerve injury (Fig. 7G).

## Discussion

In the present study, we established a new Tg mouse line in which mitochondria are labelled by GFP and *cre* recombinase is expressed specifically in injured neurons. Using this unique BAC Tg mouse, we found that mitochondrial dynamics was spatially and temporally regulated following nerve injury and that mitochondrial fission was necessary for this response, particularly at the initial stage after nerve injury. Injury-inducible *Drp1*-deficient mice demonstrated that acute and transient mitochondrial fission activation in the early phase after neuronal insult is important to maintain neuronal and mitochondrial integrity.

*Atf3*:BAC Tg mice enable highly efficient expression of exogenous genes specifically in injured neurons; in previous studies we were not able to achieve this using a virus-mediated gene transfer approach^3^,^4^. Using BAC technology, our strategy enables a specific exogenous gene to be expressed in a similar manner to that of the endogenous *Atf3* gene whose expression is markedly induced in neurons in an injury-specific manner^5^,^31^ The advantage of the *Atf3*:BAC Tg mouse is that mitochondria are GFP labelled only in injured neurons, allowing clear identification and visualisation of their cell bodies, dendrites and axons, even in living animals. Another use of the BAC Tg mouse is as an injury-inducible *Cre* driver line. By crossing with gene-floxed mice, injury-inducible gene knockout is achieved. These mice avoid developmental lethality and permit investigation of an ablated gene immediately after injury without affecting developmental or pre-injury neuronal functions. Abnormal mitochondrial dynamics are likely to be pathogenic in several neurodegenerative diseases and traumatic injuries^12^,^14^,^15^,^42^; therefore, the BAC Tg mouse will be a powerful tool for expanding our understanding of the molecular basis of damaged neurons prior to neurodegeneration.

The rapid induction of exogenous genes in the *Atf3*:BAC Tg mouse allowed us to demonstrate that mitochondria are capable of changing their dynamics promptly in response to nerve injury. Mitochondria in the soma, which are newly labelled by GFP after nerve injury, appeared shorter in injured axons. In contrast, mitochondria pre-existing in axons before injury, which are not labelled by GFP, were relatively longer. We assume that GFP-labelled shorter mitochondria are transported from the soma and that pre-existing longer mitochondria are replaced by GFP-labelled mitochondria promptly after nerve injury. We cannot rule out another possibility, that of mitochondria fusing to each other in axons, thereby transmitting GFP throughout the mitochondrial network in axons during the early stage after injury. However, a previous study has showed that mitochondria encounter each other in neurites but rarely fuse^43^, suggesting that the shorter GFP-labelled mitochondria in the soma are transported to injured axons. Moreover, the transport velocity of GFP-labelled shorter mitochondria in injured axons was increased following nerve injury, consistent with a previous study^38^. In contrast, the transport velocity of *Drp1*-deficient longer mitochondria gradually decreases. Considering that the half-life of mitochondria is reported to be around 30 days^44^, shorter mitochondria seem to be beneficial for accelerating mitochondrial exchange during the early stage after nerve injury.

At least two types of fragmented mitochondria appear to exist in damaged axons: physiologically fissioned mitochondria and contracted mitochondria. We assume that discrimination of the two types of fragmented mitochondria is crucial for understanding physiological and pathological responses in damaged neurons. The contracted mitochondria occur at injury sites, change redox balance and propagate the death signal along the axon^45^. In our study, shorter mitochondria did not appear in *Drp1*-deficient injured axons. We conclude that shorter mitochondria after nerve injury derive from divided mitochondria.

The longer mitochondria in *Drp1*-deficient motor axons were in the normal physiological range at 3 days after nerve injury because the membrane potential was maintained. During the subsequent few days, the transport velocity and membrane potential of these mitochondria then gradually decreased, although the injured neurons appeared healthy. Even though mitochondrial quality is maintained at 3 days after injury, the integrity of injured motor neurons seemed to be affected by *Drp1* deficiency because microglial cells in the proximity of *Drp1*-deficient injured motor neurons are hyper-activated. Although the mitochondrial membrane potential was maintained, it is possible that the change in mitochondrial shape during the early stages after nerve injury was due to altered intracellular and extracellular signals^46^,^47^. Collectively, mitochondrial fission, at least during the early phase after injury, could be beneficial for injured axons and neurons to respond to high-energy demand in growth cones and branching points as well as to preserve the integrity of injured motor neurons^48^.

Mitochondrial fission is generally considered to make cells susceptible to death; however, the balance between fusion and fission is likely to be important. Both *Mfn2*-deficiency and *Drp1*-deficiency resulted in degeneration of Purkinje cells in a similar manner^22^,^49^. In line with this, our study suggests that acute mitochondrial fission in response to nerve injury is necessary for injured motor neurons to adapt emergency situation. In this model, it would be also important that the imbalance of fission/fusion in injured motor neurons be returned to basal levels by the intermediate or late stage of the regeneration process. The duration of the balance shift toward fission required for the adaptive response may depend on the extent of damage or cellular context.

The importance of mitochondrial fission during the early phase after nerve injury is supported by injury-induced *Drp1*-deficient motor neurons suffering prompt death and axonal degeneration during 14 days after nerve injury. *Drp1*-deleted non-injured motor neurons, in which cre recombinase was introduced by adenovirus, looked healthy during 14 days after virus infection, although it might be hard to compare the data exactly with different types of cre-mediated systems. We assume that *Drp1*-deficient motor neurons without injury may require a longer time to degenerate, in a similar manner to *Mfn2*- or *Drp1*-deficient Purkinje cells^22^,^49^. In this context, the balance shift toward mitochondrial fission soon after nerve injury would be necessary for injured motor neurons. We cannot rule out the possibilities that ablation of *Drp1* affects basal metabolism to accelerate neuronal death and that DRP1 has other functions beyond mitochondrial fission, which affect mitochondrial and neuronal integrity after nerve injury. Further studies are warranted to understanding the exact role of mitochondrial fission in damaged neurons.

Mitochondrial fission is also considered to be closely associated with mitochondrial quality control^50^. We observed abnormal gigantic mitochondria in *Drp1*-deficient injured motor neurons, which indicated abnormal mitochondrial quality and was consistent with previous observations in which *Drp1* was ablated developmentally in neurons^21^,^22^. From this study we are unable to conclude the exact molecular mechanism involved in mitochondrial quality control in injured motor neurons. However, the Parkin-p62 dependent mitochondrial degradation system seems to be partly involved in the mitochondrial quality system in injured motor neurons. Interestingly, motor neurons in an ALS model are susceptible to proteasome degradation but not to the autophagy-lysosome system^51^,^52^. In line with this, we assume that a proteasome-dependent mechanism is involved in mitochondrial degradation in injured motor neurons (our unpublished data). Furthermore, a specialised mitochondrial degradation system is reported in axons where acidified vesicles, such as autophagosomes and late endosomes, contain damaged mitochondria^53^. These mitochondrial quality control systems may also be deregulated in *Drp1*-deficient injured motor neurons and axons.

In conclusion, the present study provides *in vivo* evidence that mitochondrial dynamics is spatially and temporally regulated in response to nerve injury, and demonstrates the possibility that acute mitochondrial fission is rather adaptive response to neuronal damage. The duration and degree of mitochondrial fission may affect neuronal and axonal protection and may depend on cell type or cellular context. Further understanding of mitochondrial dynamics *in vivo* spatially and temporally is necessary for its assessment under pathological conditions, and its appropriate manipulation has promise in preventative and therapeutic strategies for the treatment of neurodegenerative diseases and traumatic injuries.

## Methods

### Animals

All animal protocols were carried out in accordance with “the University Animal Committee Guidelines for the Care and Use of Laboratory Animals”, and were approved by the Nagoya University Institutional Animal Care and Use Committee. All possible efforts were made to minimize suffering.

*Atf3*:MitoGFP-Cre BAC (*Atf3*:BAC) Tg mice were generated according to general BAC modification protocols. The BAC clone RP24-318C6, containing the *Atf3* locus, was purchased from the BACPAC Resources Center at the Children’s Hospital Oakland Research Institute (CHORI) (BPRC, California, USA). Briefly, a gene fragment of GFP from *Aequorea coerulescens* (AcGFP) attached to the mitochondrial targeting sequence of cytochrome c oxidase subunit VIII, followed by an IRES, cre recombinase and SV40-polyA, was inserted into PL451 in front of the FRT-Neo-FRT cassette. Appropriate 5′ and 3′ 500 bp homology arms were designed immediately upstream of the ATG initiation codon and shortly downstream of the 2^nd^ exon carrying the ATG initiation codon, respectively. For recombineering, the BAC targeting cassette was excised by restriction digestion, purified to approximately 100 ng/μl, and electroporated into competent SW105 cells containing the RP24-318C6 BAC clone. After neo deletion, BAC DNA was purified and injected into pronuclei from BDF1 (DBA/2:C57BL/6) oocytes (SLC, Hamamatsu, Japan) and C57BL/6 oocytes (Institute of Immunology Co., Ltd., Tochigi, Japan) to generate Tg mice. Three lines of founder BAC Tg (*Atf3*:BAC Tg) mice (two males and one female) obtained and crossed with C57BL/6Ncr mice for at least seven generations. Genotypes were determined by polymerase chain reaction (PCR) using the following primers; forward 5′-AGAAAGCAGCACTTCCCAGAAGTCTCC-3′ and reverse 5′-AGCAGCAGCGGCGTCAGGACGGACATG-3′. Three lines of hemizygous founder mice were fertile and viable without neurological abnormalities. Drp1^*flox/flox*^ mice, originally generated by Ishihara *et al*., were provided by Ishihara T, Nomura M and Mihara K (Kyushu University) and crossed with three lines of BAC Tg (*Atf3:*BAC Tg) mice. Three lines of BAC Tg mice crossed with Drp1^*flox/flox*^ mice showed similar phenotype. Among them, one line of BAC Tg mice crossed with Drp1^*flox/flox*^ mice was used for this study.

### Plasmid

To examine whether the BAC fragment carrying MitoGFP, IRES, Cre recombinase and SV40-polyA (MitoAcGFP-Cre) is functional, the fragment was subcloned into pBluescript, in which CMV promoter sequence was inserted in front of the fragment (pCMV-MitoAcGFP-Cre). The plasmid was co-transfected into COS-7 cells together with pDsRed2-Mito vector (Takara, Shiga, Japan) and confirmed that GFP signal was localized in mitochondria. To examine the activity of cre recombinase, Adenovirus carrying loxp-neo-loxp-LacZ (AxCALNLNZ)^4^ was co-infected in COS-7 cells together with the pCMV-MitoAcGFP-Cre plasmid. After 48 h of transfection, COS-7 cells were incubated with X-gal (Roche, Basel, Switzerland) and the activity of ß-galactosidase was verified. The cell lysate of COS-7 cells expressing pCMV-MitoAcGFP-Cre plasmid was used as a positive control for western blotting.

### Surgical procedures

Animals of either sex at 6–9 weeks of age were anesthetized with pentobarbital (45 mg/kg). For sciatic nerve injury, a small incision was made on the skin. The right sciatic nerve was exposed in the mid-thigh, removed from connective tissue and cut with a pair of scissors. The incision was closed with nylon suture. For hypoglossal nerve injury, the right hypoglossal nerve was carefully exposed under the digastric muscle and transected with a pair of scissors. For optic nerve injury, the right optic nerve was carefully exposed with a small inscision. The nerve was crushed with forceps (Dumoxel #5/45 forceps, Dumont) for 5 s at a point ~1 mm behind the optic disk.

### Immunohistochemical staining

Mice of either sex were perfused transcardially with Zamboni solution. Brains and proximal region of sciatic nerves (approximately 1.5–1.8 cm from cell body) were removed, post-fixed and sectioned or teased. The sectioned samples were blocked with 1% BSA/0.3% Triton in 0.01 M PBS and incubated with primary antibody: anti-GFP, anti-ATF3, anti-cytochrome c, anti-DRP1, anti-Iba1, anti-GFAP, anti-p62 or anti-Parkin overnight at 4 °C or with anti-ChAT antibody overnight at room temperature.

Sciatic nerve fibers were teased, permeabilized in Triton X-100 for 1 hr, and washed in 0.01 M PBS. Nerve fibers were blocked with 3% NGS/10% Triton in 0.01 M PBS and incubated with primary antibodies including anti-GFP and anti-cytochrome c for 2 days and nights at 4 °C, washed, and then incubated with secondary antibody. Nerves were washed in PBS, placed on slides and mounted.

Images were acquired on a confocal laser scanning microscope (Olympus FV10i, Tokyo, Japan) using a ×60 water-immersion objective (NA1.2) or on a fluorescence microscope (Olympus BX53, Tokyo, Japan) using a ×20 objective (NA 0.70). Number of mitochondria per 10 μm^2^ of axons was counted in *Atf3*:BAC Tg (control; 942 cytochrome c-positive mitochondria in four mice, injured; 1353 GFP-positive mitochondria in six mice) and Drp1 CKO mice (control; 481 cytochrome c-positive mitochondria, injured; 1035 mitochondria from four-seven mice, respectively). Number and immunoreactive intensity of spinal motor neurons were counted and quantified, while GFP-positive neurons were identified as injured spinal motor neurons. For total area of microglial cells in 150 μm^2^, 30–50 areas from four mice were quantified.

### Immunoblotting

Mouse tissues were dissected and homogenized in lysis buffer containing 150 mM NaCl, 20 mM Tris-HCl (pH 7.5), 10 mM EDTA, 1% NP-40, 0.5% deoxycholate, 0.1% SDS, 5 μg/mL aprotinin, 1 mM PMSF, and 1 μg/mL leupeptin, and centrifuged to collect the supernatant. Then 60 μg of protein was resolved by SDS-PAGE and processed for western blot analysis. Anti-GFP and anti-GAPDH primary antibodies and were diluted 1:1000–1:5000. Signals were detected using ECL (GE Healthcare, Chalfont St Giles, UK).

### Counting of surviving motor neurons

For quantification of cell survival after axotomy, sections were stained with thionine. Thionine-stained motor neurons in injured and control sides were counted separately and at the identical level between animals as previously described^8^. Data are presented as the percentage of surviving neurons on the injured and control side (50–70 sections from eight mice per group). Statistical significance (*p* value) was calculated by two-tailed Student’s *t* test.

### *In vivo* imaging

Mice of either sex were anesthetized by i.p. injection of pentobarbital (45 mg/kg). To image axonal mitochondria in the sciatic nerve, we surgically exposed the sciatic nerve without causing damage, and positioned the mouse on an inverted laser-scanning confocal microscope (Olympus FV10i). For *in vivo* time-lapse imaging, we followed GFP-labelled mitochondria in the nerve. Injured sciatic nerves were imaged using a ×60 water-immersion objective (NA1.2). Time lapse images of mitochondrial dynamics were collected in a single focal plane at 1024 × 1024 pixel resolution every 5 sec for a total of 200 images.

To measure the electrochemical potential of mitochondria *in vivo*, the potential-sensitive dye TMRM (Life Technologies, Carlsbad, CA, USA) was applied to the surgically exposed sciatic nerve at a concentration of 1.25 μM diluted in PBS (25 mM DMSO stock) and incubated for 30 min in the dark. Excess dye was removed by repeated washing with saline. Mitochondrial labeling disappeared immediately after application of 100 μM of a mitochondrial uncoupling reagent, carbonyl cyanide *m*-chlorophenyl hydrazine (CCCP) (Sigma-aldrich, St. Louis, USA) Sciatic nerves were imaged using a ×60 water-immersion objective (NA1.2).

### Quantification of mitochondria

Morphological features of mitochondria were imaged with a confocal laser scanning microscope (Olympus FV10i) using a ×60 objective (NA1.2). For imaging mitochondrial morphological features, a serial z stack of 0.3 or 0.5 mm was acquired with a digital zoom of 4 or 5 (×60 objective). Regions of interest corresponding to the shape of individual mitochondria were manually drawn. The obtained number of pixels was converted to micrometers, and the distribution of their length was shown on a graph against their frequency. The number of mitochondria was counted in a given area of axons from mice. Control; 942 cytochrome c-positive mitochondria in four mice, 7 d; 1038 cytochrome c-positive and 1353 GFP-positive mitochondria in six mice, 56 d; 451 cytochrome c-positive and 443 GFP-positive mitochondria in four mice. To determine the length, number and velocity of motile mitochondria, kymographs were generated using the Image J software (NIH, Bethesda, MD, USA) as described previously^13^. Motile mitochondria appeared as diagonal lines, and their slopes provided velocity. Each mobile mitochondrion confirmed to be moving for six or more consecutive frames within the area was measured. Average speed of moving mitochondria (the displacement during runs divided by the duration of the run) was defined as mitochondrial velocity (200–300 mitochondria from four and five mice in each group, respectively). The number of moving mitochondria per minute that crossed a vertical line of a single axon was counted and defined as number of moving mitochondria in 20–50 axons from four mice. To evaluate the mitochondrial potential, we measured the fluorescence intensity of individual 150–350 TMRM-labeled mitochondria from sciatic nerves of three to five mice. Stationary mitochondrial sites were identified as vertical lines on the kymographs and counted per area.

### Statistical analysis

Data were analyzed for normal distribution and equal variance. Significance was determined using Student’s *t-*test. Significance levels are indicated as *p* < 0.001. A one-way ANOVA was performed for multiple comparisons with Turkey post hoc analysis as *p* < 0.001. The number of animals used in each experiment was included in the figure legends.

## Additional Information

**How to cite this article**: Kiryu-Seo, S. *et al*. Mitochondrial fission is an acute and adaptive response in injured motor neurons. *Sci. Rep.* **6**, 28331; doi: 10.1038/srep28331 (2016).

## Supplementary Material

Supplementary Information

Supplementary Movie 1

Supplementary Movie 2

Supplementary Movie 3

Supplementary Movie 4
