# Supplementary material for: Mitochondrial fission is an acute and adaptive response in injured motor neurons
Source: Sci Rep. 2016 Jun 20;6:28331. doi: 10.1038/srep28331 (PMC4913268; doi:10.1038/srep28331)
Supplement: Supplementary Information [file srep28331-s1.pdf]

Supplementary information

Mitochondrial fission is an acute and adaptive response in injured motor neurons

Sumiko Kiryu-Seo[1,2,*], Hiromi Tamada[1], Yukina Kato[1], Katsura Yasuda[1], Naotada Ishihara[3], Masatoshi Nomura[4], Katsuyoshi Mihara[5], Hiroshi Kiyama[1,2,*]

[1]Department of Functional Anatomy and Neuroscience, Graduate School of Medicine, Nagoya University, 65 Tsurumai-cho, Showa-ku, Nagoya 466-8550, Japan

[2]CREST, JST, 65 Tsurumai-cho, Showa-ku, Nagoya 466-8550, Japan

[3]Department of Protein Biochemistry, Institute of Life Science, Kurume University, Kurume 839-0864, Japan

[4]Department of Medicine and Bioregulatory Science and [5]Department of Molecular Biology, Graduate School of Medical Science, Kyushu University, Fukuoka 812-8382, Japan

***Corresponding authors**

Sumiko Kiryu-Seo and Hiroshi Kiyama

E-mail: skiryu@med.nagoya-u.ac.jp and kiyama@med.nagoya-u.ac.jp

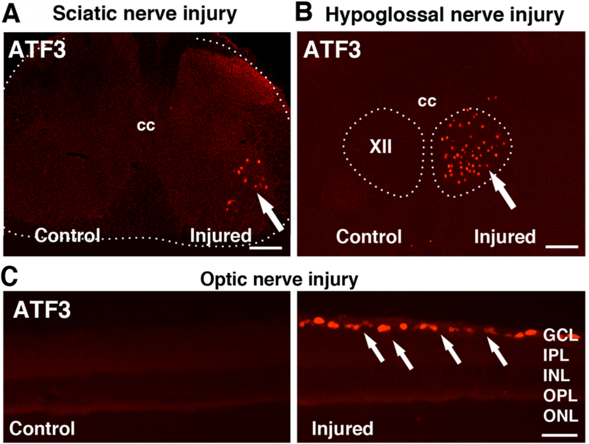

Kiryu-Seo et al._Supplementary Fig. S1

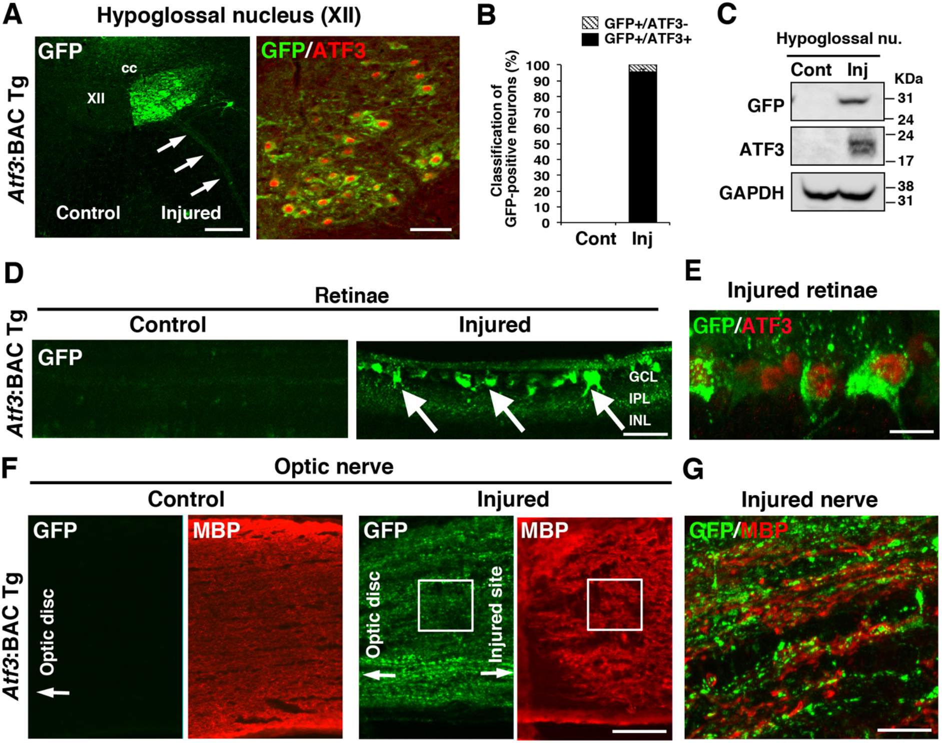

Kiryu-Seo et al._Supplementary Fig. S2

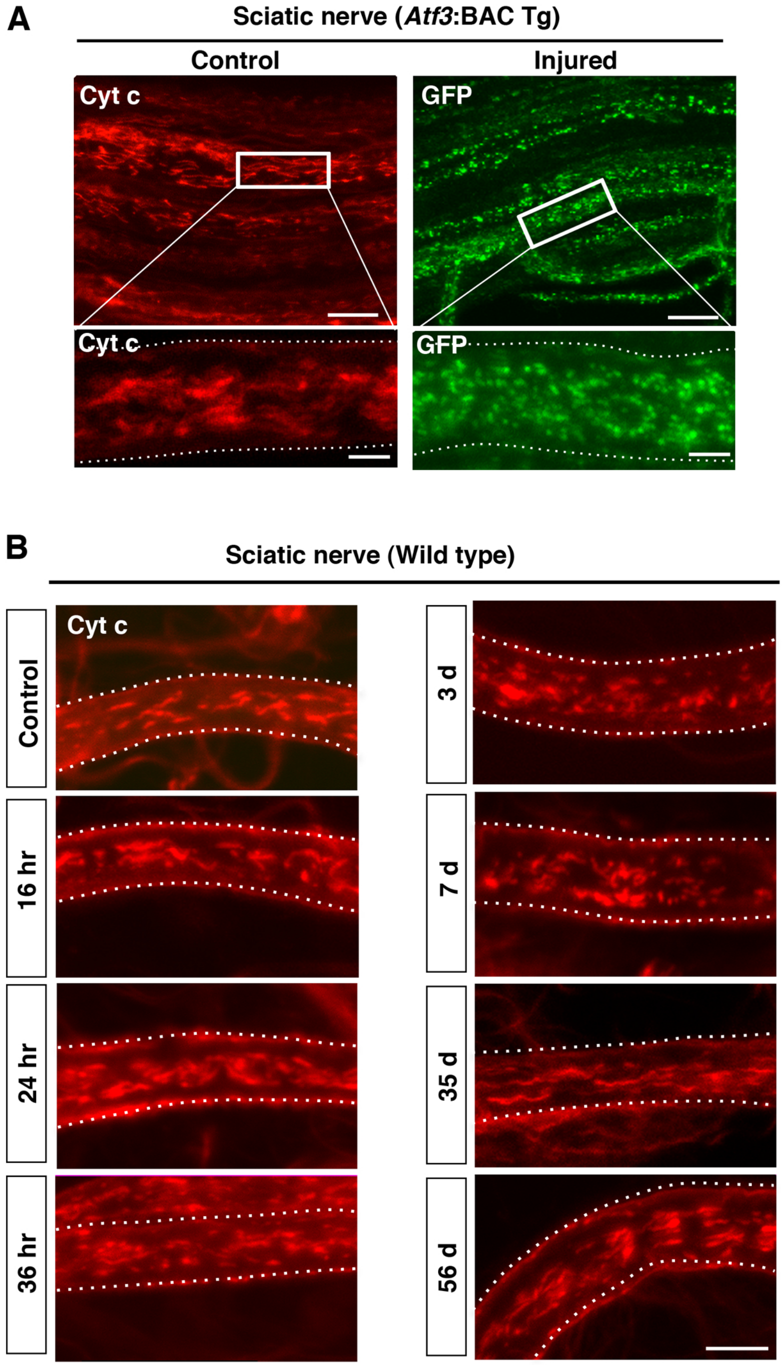

Kiryu-Seo et al._Supplementary Fig. S3

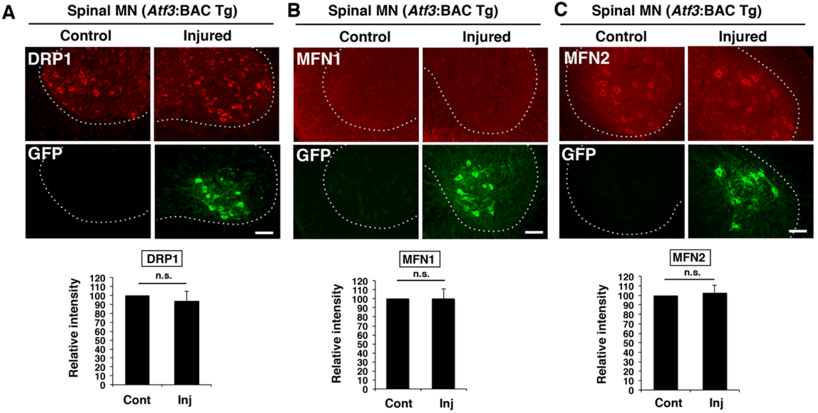

Kiryu-Seo et al._Supplementary Fig. S4

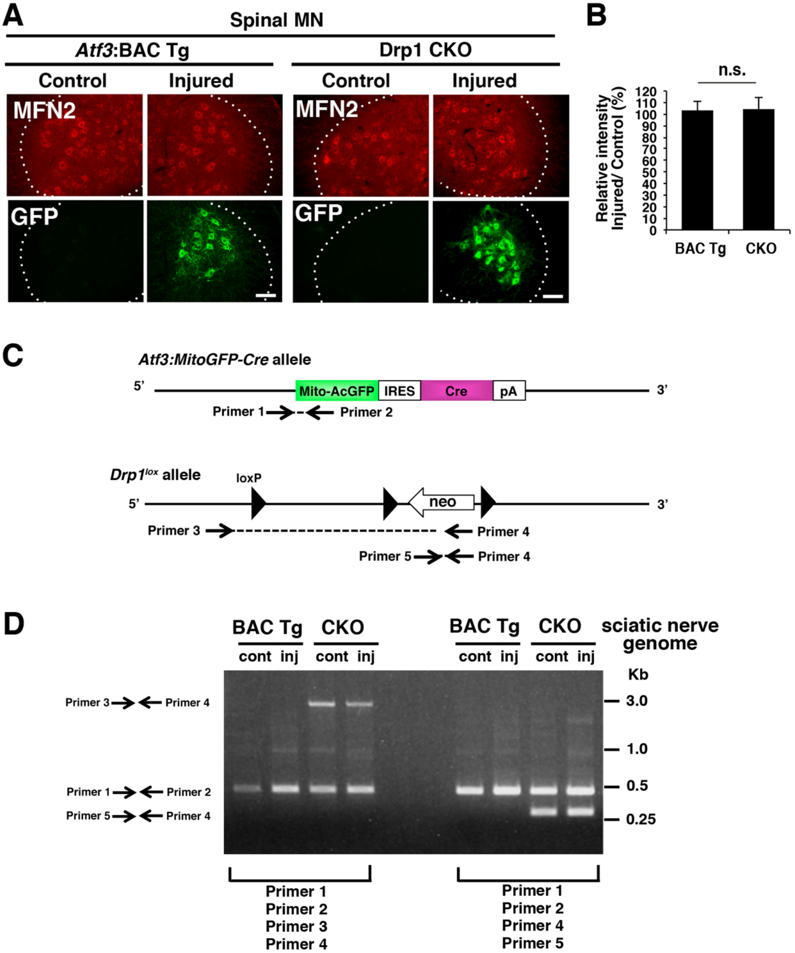

Kiryu-Seo et al._Supplementary Fig. S5

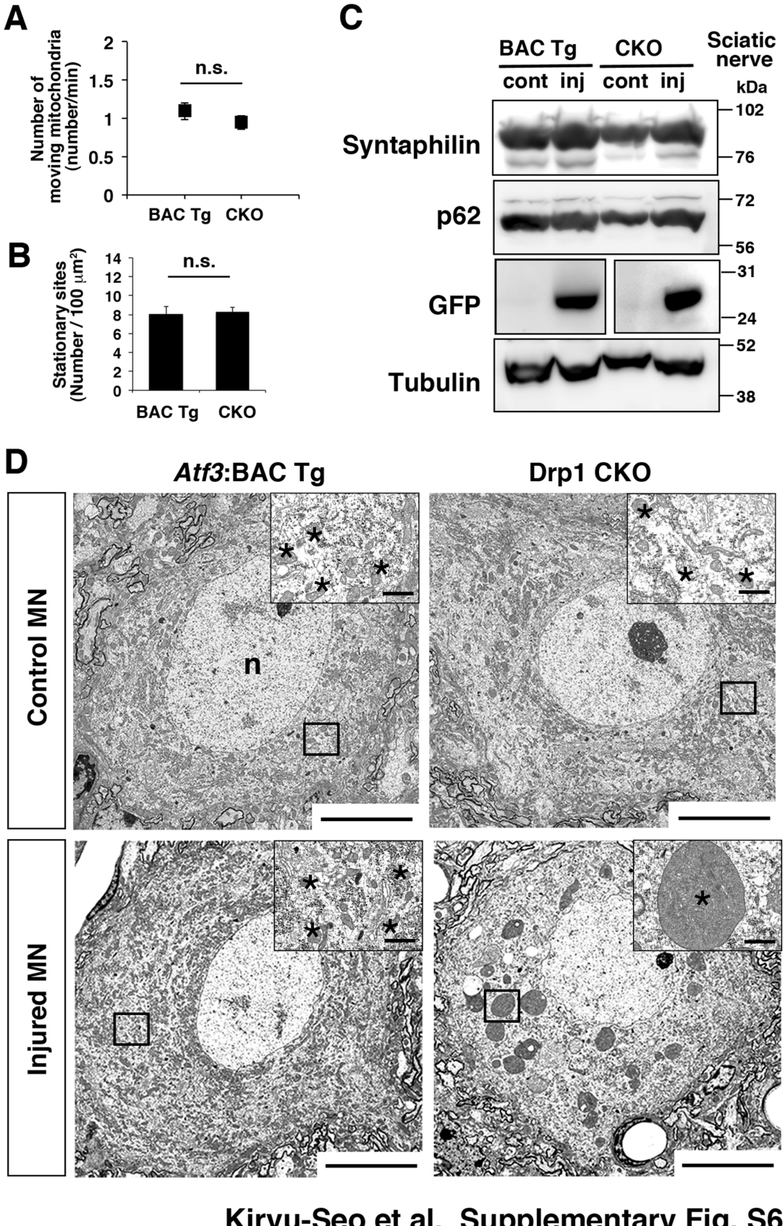

Kiryu-Seo et al._Supplementary Fig. S6

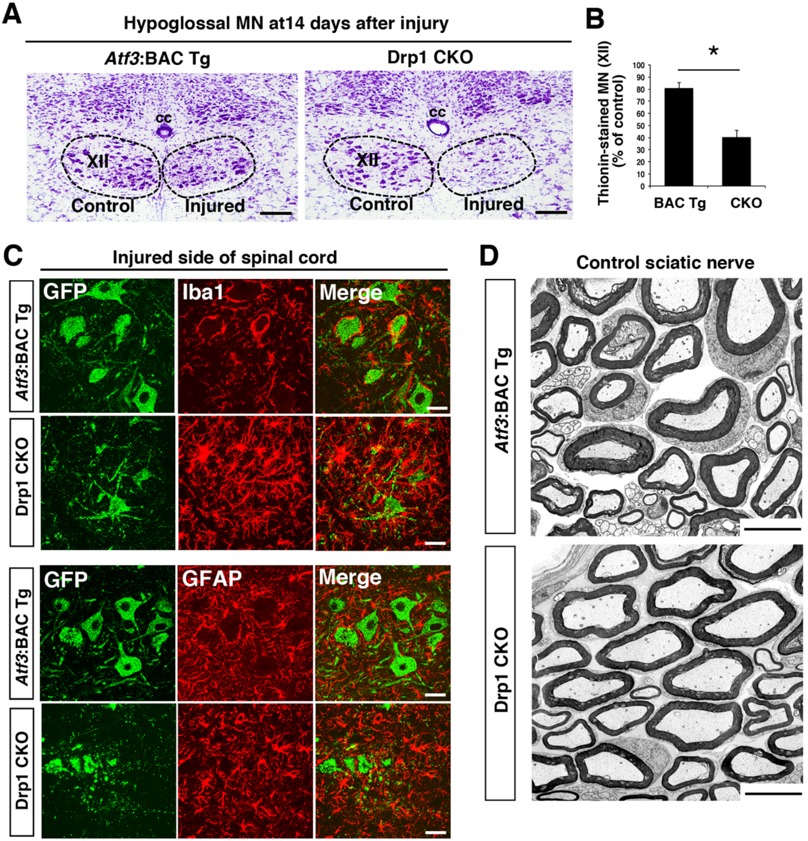

Kiryu-Seo et al._Supplementary Fig. S7

Supplementary Figure Legends

Supplementary Fig. S1 Expression of ATF3 following nerve injury

(A) Sciatic nerve injury and (B) hypoglossal nerve injury induced the expression of ATF3 at 7 days after injury. (C) Optic nerve injury induced expression of ATF3 in injured retinal ganglion cells at 3 days after injury (arrows). Scale bars, 200 μm in (A and B) and 40 μm in (C).

Supplementary Fig. S2 Expression of GFP following hypoglossal and optic nerve injuries

(A) *Atf3*:BAC Tg (*Atf3*:MitoAcGFP-Cre) mice showed induced expression of GFP in injured hypoglossal nucleus at 7 days after injury (left). Arrows indicate injured hypoglossal nerve. GFP-positive hypoglossal motor neurons express ATF3 at 7 days after hypoglossal nerve injury (right). (B) Population of immunoreactive cells in control and injured side of hypoglossal nucleus. (C) Immunoblot of GFP and ATF3 in control and injured hypoglossal nuclei at 7 days after nerve injury. (D) *Atf3*:BAC Tg mice showed induced expression of GFP in retinal ganglion cells (arrows) at 3 days following optic nerve injury. (E) GFP-positive retinal ganglion cells after optic nerve injury also expressed ATF3. (F) Immunohistochemical staining of GFP and MBP using control and proximal site of injured optic nerve at 3 days after optic nerve crush injury. (G) High-magnification of proximal site in injured optic nerve stained by GFP and MBP. Scale bars, 200 μm in (A, left), 50 μm in (A, right), 40 μm in (D), 10 μm in (E) and 60 μm in (F) and 20 μm in (G). cc, central canal; XII, hypoglossal nucleus; GCL, retinal ganglion cell layer; IPL, inner plexiform layer; INL, inner nuclear layer.

Supplementary Fig. S3 Mitochondrial appearance after sciatic nerve injury

(A) Representative images of teased sciatic nerves from the control and injured sides at 7 days after injury. The control and injured nerves are stained for Cyt c and GFP, respectively. The area surrounded by the white box in the upper panel is magnified in the lower panel. The lower panel is a high-power magnified image of a single axon shown by the white dotted line. (B) Time-dependent morphology of cytochrome c (Cyt c)-stained mitochondria in axon before and after sciatic nerve injury. Dashed lines show the outline of a single axon. Scale bars, 10 μm in (A, upper), 2.5 μm in (A, lower) and 5 μm in (B).

Supplementary Fig. S4 Expression of proteins related to mitochondrial fission and fusion after sciatic nerve injury

Immunohistochemical staining for DRP1 (A), MFN1 (B) and MFN2 (C) in control and injured spinal motor neurons at 7 days after sciatic nerve injury. GFP-positive neurons indicate injured motor neurons. Signal intensity of immunoreactivity for DRP1, MFN1 and MFN2 in control and injured GFP-positive motor neurons was quantified. Data are means ± SEM compared with the contralateral side. n = 3 mice for each group, n.s. not significant. Scale bars, 100 μm (A-C).

Supplementary Fig. S5 Nerve injury-induced *Drp1* ablation

(A) Immunohistochemical staining of MFN2 in control and injured motor neurons at 3 days after nerve injury in *Atf3*:BAC Tg and Drp1 CKO mice. (B) The immunoreactive intensity of MFN2 in control and GFP-positive injured motor neurons was quantified. Data are presented as the percentage compared with that of control neurons (mean ± SEM, n = 3 mice in each group, n.s. not significant). Scale bar, 100 μm. (C) Schematic diagram of constructs. Arrowheads indicate loxP sequence. The arrows indicate primers used for genotyping by PCR. (D)

Identification of floxed *Drp1* and *MitoGFP-Cre* by PCR genotyping using sciatic nerve. The majority of the DNA extracted from sciatic nerve was derived from genome of Schwann cells but not from neurons. The primer pairs (arrows) of 1 and 2 identify *Atf3*:BAC Tg, while primer pairs of 3 and 4 or 4 and 5 identify the floxed *Drp1* gene that is not deleted by cre recombinase. The genotyping using sciatic nerves revealed that *Drp1* was not ablated in either control or injured sciatic nerves of Drp1 CKO mice. BAC Tg; *Atf3*:BAC Tg mice, CKO; Drp1 conditional knockout mice,

Supplementary Fig. S6 *Drp1* deficiency specifically in injured motor neurons causes the alteration of mitochondrial integrity

(A) The number of mitochondria transported in a single axon in normal sciatic nerve. Data are means ± SEM. (n = 3 mice respectively, n.s. not significant). Mitochondria were labelled by TMRM before measurement. (B) The number of stationary mitochondrial sites per 100 μm^2 area in sciatic nerve. Values are means ± SEM (n = 4 mice, n.s. not significant). Mitochondria were labelled by TMRM before measurement. (C) Immunoblotting of syntaphilin, p62/SQSTM1, GFP and tubulin using control and injured sciatic nerves at 7 days after injury. (D) Electron micrograph using x 1,200 objective of control and injured motor neurons in *Atf3*:BAC Tg and Drp1 CKO mice. The area surrounded by a black box was magnified in inset. n; nucleus, Asterisk shows mitochondria. BAC Tg; *Atf3*:BAC Tg mice, CKO; Drp1 conditional knockout mice, Scale bars, 10 μm in (D) and 1 μm in (D, inset).

Supplementary Fig. S7 Injury-induced *Drp1*-deficient mice show motor neuron death promptly after nerve injury

(A) Thionin staining of hypoglossal motor neurons at 14 days after hypoglossal nerve injury in *Atf3*:BAC Tg and Drp1 CKO mice. Dashed lines denote hypoglossal nucleus (XII). cc; central canal. (B) Percentage of surviving motor neurons in *Atf3*:BAC Tg and Drp1 CKO mice at 14 days after hypoglossal nerve injury. Data are means ± SEM. n = 8 mice per group, $^{*}p < 0.001$. (C) Expression of Iba1 (red) and GFAP (red) surrounding injured spinal motor neurons (green). Note that the expression of GFAP did not differ between *Atf3*:BAC Tg and Drp1 CKO mice, unlike that of Iba1. (D) Electron micrograph using x 3,000 objective of control sciatic nerve in *Atf3*:BAC Tg and Drp1 CKO mice. Scale bars, 100 µm in (A and C) and 5 µm in (D).

Supplementary movies

Supplementary Movie S1: *In vivo* time-lapse images of GFP-labeled mitochondria in the proximal region of an injured sciatic nerve of *Atf3*:BAC Tg mouse at 3 days after injury. Time-lapse image analysis was performed using laser-scanning confocal microscopy (Olympus FV10i). Images were recorded every 5 s.

Supplementary Movie S2: *In vivo* time-lapse images of GFP-labeled mitochondria in the proximal region of an injured sciatic nerve of the Drp1 CKO mouse at 3 days after injury. Time-lapse image analysis was performed using laser-scanning confocal microscopy (Olympus FV10i). Images were recorded every 5 s.

Supplementary Movie S3: *In vivo* time-lapse images of GFP-labeled mitochondria in an injured sciatic nerve of *Atf3*:BAC Tg mouse at 7 days after injury. Time-lapse image analysis was performed using laser-scanning confocal microscopy (Olympus FV10i). Images were recorded every 5 s.

Supplementary Movie S4: *In vivo* time-lapse images of GFP-labeled mitochondria in an injured sciatic nerve of the Drp1 CKO mouse at 7 days after injury. Time-lapse image analysis was performed using laser-scanning confocal microscopy (Olympus FV10i). Images were recorded every 5 s.

Supplementary Methods

Antibodies

Sources of antibodies were as follows: polyclonal anti-GFP (MBL International, Cat# 598, RRID:AB_591819), monoclonal-GFP (Millipore (Chemicon / Upstate / Linco, Cat# MAB2510, RRID:AB_94623), anti-cytochrome c (Invitrogen, Cat# 456100, RRID:AB_1500538), anti-GAPDH (Ambion, Cat# AM4300, RRID:AB_437392), anti-ATF3 (Santa Cruz Biotechnology, Cat# sc-188, RRID:AB_2258513), anti-MBP (Covance, Cat# SMI-94R-100, RRID:AB_10123764 and Cat# SMI-99P-100, RRID:AB_10120129), anti-choline acetyltransferase (ChAT) (Millipore (Chemicon / Upstate / Linco), Cat# AB144P-1ML, RRID:AB_262156), anti-DRP1 (Cell Signaling Technology, Cat# 8570S, RRID:AB_10950498), anti-Parkin (R&D Systems, Cat# AF1438, RRID:AB_2267901), anti-Iba1 (Wako Chemicals USA, Cat# 016-20001, RRID:AB_839506), anti-CD11b (AbD Serotec Cat# MCA711GT, RRID:AB_1100616) and anti-GFAP (Sigma-Aldrich, Cat# G3893, RRID:AB_477010), anti-p62 (ARP American Research Products Cat# 03-GP62-C, RRID:AB_1542690), anti-syntaphilin (Santa Cruz Biotechnology Cat# sc-33824, RRID:AB_2193559), anti-mitofusin-1 (Abcam Cat# ab57602, RRID:AB_2142624), anti-mitofusin-2 (Cell signaling #9482) and anti-βIII tubulin (Promega Cat# G7121, RRID:AB_430874).

PCR genotyping of sciatic nerve

Sciatic nerves were collected and incubated in lysis buffer containing 1 × SSC, 10 mM Tris-HCl (pH 7.5, 1 mM EDTA, 1% SDS and 200 μg/ml proteinase K) at 50°C overnight and genomic DNA was extracted using standard phenol/chloroform extraction. The obtained genomic DNA was used as a template for PCR genotyping. Primer sequences were as follows: primer 1; 5′-

AGAAAGCAGCACTTCCCAGAAGTCTCC-3′, primer 2; 5′-AGCAGCAGCGGCGTCAGGACGGACATG-3′, primer 3; 5′-CAGCTGCACTGGCTTCATGACTC-3′, primer 4; 5′-AAGCTCTTCAGCAATATCACGGGTAGC-3′, primer 5; 5′-GCCCATTCGACCACCAAGCGAAACATC-3′.

Immunoblotting

Mouse sciatic nerves were dissected and homogenized in lysis buffer containing 150 mM NaCl, 20 mM Tris-HCl (pH 7.5), 10 mM EDTA, 1% NP-40, 0.5% deoxycholate, 0.1% SDS, 5 μg/mL aprotinin, 1 mM PMSF, and 1 μg/mL leupeptin, and centrifuged to collect the supernatant. Then 60 μg of protein was resolved by SDS-PAGE and processed for western blot analysis. Primary antibodies used were anti-GFP, anti-syntaphilin, anti-p62 and anti-βIII tubulin.

RNA isolation and quantitative reverse-transcription PCR

Total RNA was extracted from tissues using an RNeasy Lipid Tissue kit (Qiagen, Hilden, Germany) according to the manufacturer's instructions. cDNA was synthesized using SuperscriptIII (Invitrogen) following the standard protocols. Quantitative PCR was performed on the StepOnePlus™ Real-Time PCR System using primer pairs and SYBR Green PCR Master Mix (Life technologies). Data were normalized to the GAPDH levels and calculated using the comparative threshold cycle method. The sequences of primers used are as follows; GFP forward 5'- AGTTCATCTGCACCACCG -3', reverse 5'- AAGTCGTGCTGCTTCATGTG -3'; GAPDH forward 5'- ACTCCACTCACGGCAAATTCA -3', reverse 5'- TCTCGCTTCTGGAAGATGGT 3'.

Electron microscopy

Mice of either sex were perfused with 3% glutaraldehyde and 4% paraformaldehyde in 0.1 M phosphate buffer. The tissue was placed in 3% glutaraldehyde and 4% paraformaldehyde in 0.1 M cacodylate buffer (pH 7.4) for 2 hr at 4°C and post-fixed in 1% osmium tetroxide in the same buffer for 2 hr at 4°C, rinsed with distilled water, block-stained overnight in a saturated solution of uranyl acetate, dehydrated in an ethyl alcohol series, and embedded in epoxy resin. Following examination of semi-thin sections stained with toluidine blue to select suitable areas, ultrathin sections were cut using an ultramicrotome (Leica Microsystems, Wetzlar, Germany), double-stained with uranyl acetate and lead citrate, and processed for observation with a transmission electron microscope (JEOL JEM 1400EX, JEOL, Tokyo, Japan).

Adenovirus injection

We constructed adenovirus carrying cre recombinase (Ad-Cre) under the control of the DINE promoter region (-2765/+1) to ensure neuron-specific expression [31]. The recombinant viruses were generated according to a previously described method [4]. The Ad-Cre was injected to the surgically exposed sciatic nerve of either wild type or Drp1$^{flox/flox}$ mouse.
